# Clinical trial participation predicts improved survival in older adults receiving allogeneic blood and marrow transplant

**DOI:** 10.1186/s12877-023-03803-7

**Published:** 2023-03-04

**Authors:** Clifton P. Thornton, Karen Bandeen-Roche, Madeline Dolinar, Laken C. Roberts Lavigne, Dina George Lansey, Rick Jones, Jeremy Walston, Ravi Varadhan, Melissa Hladek, Philip Imus

**Affiliations:** 1 Johns Hopkins School of Nursing, Baltimore, USA; 2 Children’s Hospital of Philadelphia, Philadelphia, USA; 3 Johns Hopkins Bloomberg School of Public Health, Baltimore, USA; 4 Johns Hopkins Sidney Kimmel Comprehensive Cancer Center, Baltimore, USA

**Keywords:** Older adults, Geriatric, Clinical studies, Oncology, Allogenic stem cell transplant, Health equity

## Abstract

**Background:**

Older adults represent a large oncologic demographic and are under-represented within oncology research despite constituting nearly two-thirds of the oncologic population in the United States. Because many social factors influence research participation, those who enroll in research do not reflect the oncology population at large, introducing bias and creating issue with external validity of studies. The same factors that influence study enrollment may also impact cancer outcomes, meaning that those who enroll in studies may already have an improved chance of cancer survival, further skewing results of these studies. This study evaluates characteristics that influence study enrollment in older adults and explore to what degree these factors may influence survival after allogeneic blood or marrow transplantation.

**Methods:**

This retrospective comparison study evaluates 63 adults aged 60 and above undergoing allogenic transplantation at one institution. Patients who elected and declined enrollment in a non-therapeutic observational study were evaluated. Demographic and clinical characteristics between groups were compared and assessed as predictors of transplant survival, including decision to enroll in the study.

**Results:**

Participants who chose to enroll in the parent study were not different with regard to gender, race/ethnicity, age, insurance type, donor age, and neighborhood income/poverty level compared to patients who were invited to participate but declined enrollment. The research participant group had higher proportion assessed as being fully active (23.8% vs. 12.7%, *p* = 0.034) and lower mean comorbidity scores (1.0 vs 2.47, *p* = 0.008). Enrollment in an observational study independently predicted transplant survival (HR = 0.316, 95% CI 0.12–0.82, *p* = 0.017). When controlling for relevant confounders of disease severity, comorbidities, and transplant age, enrolling in the parent study was associated with a lower hazards of death following transplant (HR = 0.302, 95% CI 0.10–0.87, *p* = 0.027).

**Conclusions:**

Despite being demographically comparable, persons who enrolled in one non-therapeutic transplant study had significantly improved survivorship than those who did not participate in observational research. These findings suggest that there are unidentified factors that influence study involvement that may also impact disease survivorship, over-estimating outcomes from these studies. Results from prospective observational studies should be interpreted with the consideration that study participants have an improved chance of survival at baseline.

## Introduction

Allogeneic blood or marrow transplantation (alloBMT) is the standard of care and sometimes the only potential cure for many hematologic malignancies. Steady advancements in the safety of alloBMT have resulted in significant improvements in morbidity and non-relapse mortality [1], but transplant continues to be an intensive intervention, especially for older adults [2]. Despite calls to improve representation of age, gender and race/ethnicity in clinical research [3], participants in oncology research remain predominantly white, young, and from higher socioeconomic backgrounds [4, 5]. This is particularly concerning with regard to older adults since persons over the age of 65 constitute 63.4% of the nation’s oncologic population and nearly 1 out of every 5 older adults have had a cancer diagnosis. Given the current understanding of social determinants of health and that persons who elect to enroll in any type of clinical study come from higher resourced backgrounds, they likely have improved health status and may, at baseline, have an improved chance of survival after treatment. Skewed study enrollment toward healthier and more well-resourced participants in clinical trials and investigative works limits application of findings to broad healthcare populations and provides little insight to the current status of all persons with cancer. Therefore, studies that drive improvements in care may be based on a biased sample and not apply to the oncology population at large. The current study evaluates factors that influence older adults’ decision to participate in an observational alloBMT study in order to identify factors that may influence study enrollment and ultimately compare differences in outcomes between those who participate and decline enrollment in a prospective non-therapeutic study.

## Methods

All patients aged 60 and above who were undergoing alloBMT for a hematologic malignancy between July 2019 and March 2020 at one academic institution were offered participation in a non-therapeutic observational study (NCT04188678). This parent study, REBOUND, evaluates physical resilience in older adults receiving a stem cell transplant and involves physical function assessments, questionnaires about general health, cognitive assessments, personality/psychological assessments, and physiologic measures of blood, saliva, glucose tolerance, heart rate variability, and MRI collected during clinical visits. The parent study does not offer any intervention and does not intend to improve personal resilience or transplant survival. Only those who could not walk independently or speak English fluently were excluded from the parent study. Demographic, clinical data, and survivorship data were collected on all participants that were approached for enrollment. Characteristics and outcomes were compared between two groups: 1) those who elected to participate in the non-therapeutic parent study and 2) those who declined enrollment in the parent study but underwent alloBMT during the same time period at the same institution. This study was approved by the Johns Hopkins Institutional Review Board (IRB# 00,279,188).

### Data collection

Gender, race/ethnicity, age at transplant, donor age, insurance type, and referral source were collected. Hematopoietic cell transplantation comorbidity index (HCT-CI) scores, which correlate with degree of comorbidities and transplant survival [6, 7], were calculated for each participant. Disease-specific risk index was determined following guidelines from the Center for International Blood & Marrow Transplant Research [8]. Scores consider diagnosis, stage, and cytogenetics to determine risk of transplant survival as low, intermediate, high, or very high and act as a standardized evaluation of survival between individual diagnoses [9]. Eastern Cooperative Oncology Group (ECOG) scale assessed performance status; scores are clinician-assigned ranging from 0–4 and included if assessed within 30 days of transplant. A score of 0 indicates the patient is fully active with no hinderance in daily physical performance, higher scores indicate worsening function and poorer transplant outcomes [10–12]; scores were dichotomized (ECOG = 0 vs ECOG > 0) for analyses. Participant addresses were geocoded and linked to census tracts using ArcMap 10.6.1 and linked to USDA Food Access Research Atlas 2019 dataset to identify urban/rural designation, poverty rate (proportion of census tract living at or below federal poverty level), and median family income. Commute distances to the treating facility were determined via Google Maps; two participants with P.O. boxes and one outside of the continental United States were excluded from neighborhood analyses. Overall survival was calculated as the number of days from transplant until death with censoring at last known date patient was alive for those still living.

### Analysis

To compare groups, Chi square analysis evaluated differences in proportions of gender, race/ethnicity, insurance type, referral status, ECOG status, urban census tract, and low-income census tract. Independent sample t-tests compared age at transplant, donor age, comorbidity index, commute distance, poverty rate, and median family income. Univariate logistic regression was used to evaluate if the degree of neighborhood poverty/income and comorbidity score impacted REBOUND enrollment.

For survival analyses, Kaplan–Meier survival curves visualized unadjusted survival based on REBOUND enrollment and log-rank tests assessed significance in differences. Multivariable survival analyses were assessed via Cox regressions by first evaluating individual factors with hypothesized clinical influence on survival including age at transplant, donor age, ECOG status, comorbidity index, disease risk index, and REBOUND enrollment. Suspected cofounders (those with *p* < 0.25) were retained as covariates in larger models.

## Results

### Differences between groups

Sixty-three eligible patients underwent alloBMT with non-myeloablative conditioning at the study institution; 33 elected to enroll and 30 declined enrollment in the pilot phase of REBOUND. Table 1 displays demographic and clinical baseline data. Participants were demographically and clinically similar with regard to gender, race, age at transplant, type of insurance, referral status for transplant, donor age, primary oncology diagnosis, and disease risk index (all *p* > 0.05). Type of transplant was different between groups (*p* = 0.045). While those who received matched unrelated and haploidentical transplants elected to enroll in REBOUND at similar proportions (6 enrolled and 5 declined for matched unrelated; 23 enrolled and 21 declined for haploidentical), no patient who received a matched related transplant (*n* = 4) enrolled in the parent study, and all of those who received a mismatched unrelated transplant (*n* = 4) elected to enroll. When examining social health factors, participants were similar with regard to distance from the hospital, proportion from urban/rural areas, proportion from low-income areas, geographic poverty rate, and geographic mean family income (all *p* > 0.05). REBOUND participants had a higher proportion of being fully active (23.8% vs 12.7%, *p* = 0.034) and mean comorbidity scores were higher for those who declined participation in REBOUND than those who enrolled (2.47 vs 1.0, *p* = 0.008).

**Table 1:** Participant demographic data

	Enrolled (*n* = 33)	Not enrolled (*n* = 30)	
Gender			
Male	19 (30.2%)	15 (23.8%)	*p* = 0.547
Female	14 (22.2%)	15 (23.8%)	
Race			
White	30 (47.6%)	24 (38.1%)	*p* = 0.404
Black	2 (3.2%)	5 (7.9%)	
Mixed/Other	1 (1.6%)	1 (1.6%)	
Age at transplant (mean years)	67.96 (SD = 4.08)	66.89 (SD = 4.03)	*p* = 0.302
Insurance type			
Private	15 (38.1%)	11 (17.5%)	*p* = 0.479
Government/Medicare	18 (28.6%)	19 (30.2%)	
Referral status			
Referred from OSH	11 (17.5%)	10 (15.9%)	*p* = 1.000
Internal referral	22 (34.9%)	20 (31.7%)	
ECOG status (*n* = 57)			
ECOG = 0	15 (23.8%)	8 (12.7%)	*p* = 0.034
ECOG > 0	12 (19%)	21 (33.3%)	
HCT-CI Score (mean)	1.0 (SD = 1.44)	2.47 (SD = 2.73)	*p* = 0.008
Primary Oncology Diagnosis			
Myelodysplastic Syndrome	5 (7.9%)	7 (11.1%)	*p* = 0.398
Acute Myeloid Leukemia	9 (14.3%)	9 (14.3%)	
Non-Hodgkin Lymphoma	10(15.9%)	3 (4.8%)	
Multiple Myeloma	1 (1.6%)	1 (1.6%)	
Myeloproliferative Neoplasm	4 (6.3%)	3 (4.8%)	
Acute Lymphoblastic Leukemia	4 (6.3%)	5 (7.9%)	
Chronic Myeloid Leukemia	0	2 (3.2%)	
Disease Risk Index Scores			
Low	1 (1.6%)	2 (3.2%)	*p* = 0.443
Intermediate	31 (49.2%)	26 (41.3%)	
High	0	1 (1.6%)	
Donor age (mean years)	32.1 (SD = 8.11)	30.2 (SD = 6.87)	*p* = 0.348
Type of Transplant			
Matched Related	0	4 (6.3%)	*p* = 0.045
Matched Unrelated	6 (9.5%)	5 (7.9%)	
Haploidentical	23 (36.5%)	21 (33.3%)	
Mismatched Unrelated	4 (6.3%)	0	
Commute distance (mean miles)	61.6 (SD = 55.6)	56.3 (SD = 26.74)	*p* = 0.644
Urban census tract			
Rural	6 (9.5%)	10 (15.9%)	*p* = 0.190
Urban	26 (41.3%)	20 (31.7%)	
Low-income census tract			
Low income	3 (4.8%)	7 (11.1%)	*p* = 0.133
Not low income	28 (44.4%)	22 (34.9%)	
Poverty rate (mean proportion)	5.52 (SD = 6.07)	7.59 (SD = 6.71)	*p* = 0.214
Median family income (mean)	128,542 (SD = 44,378)	115,756 (SD = 44,468)	*p* = 0.270

*SD* standard deviation, *OSH* outside hospital, *HCT-CI* hematopoietic cell transplant comorbidity index, *ECOG* Eastern Cooperative Oncology Group Performance Status. Poverty rate and median family income reflects census tract of primary residence, not for individual participants

Percentages reflect proportion of entire sample (*n* = 63)

### Predictors of study enrollment

Univariate logistic regression models identified that comorbidity index scores predicted REBOUND enrollment (OR 0.71, 95% CI 0.54–0.95, *p* = 0.017) such that declining enrollment in REBOUND was more likely as comorbidity scores increased. Age at transplant, neighborhood poverty rate, commute distance, performance status, and neighborhood median family income did not predict enrollment (all *p* > 0.05).

### Predictors of overall survival

**Fig. 1:**
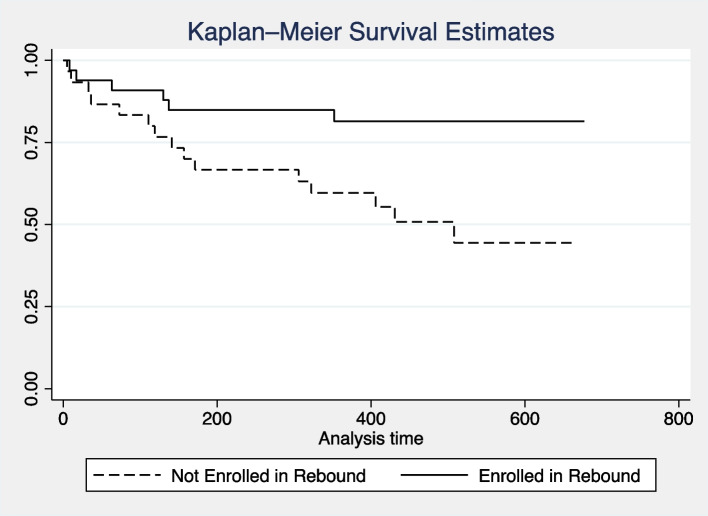
Survival estimates for REBOUND enrollment

Kaplan–Meier survival visualizations suggest that participants who elected to enroll in REBOUND had better survival following alloBMT (log-rank *p* = 0.012, Fig. 1). To evaluate how individual factors may influence survival, each clinical predictor was assessed as an independent survival predictor in Cox regression models (Table 2). Of those, only comorbidity index (HR 1.17, 95%CI 1.01–1.36, *p* = 0.032) and enrollment in REBOUND (HR 0.316, 95%CI 0.12–0.82, *p* = 0.017) were independently associated with overall survival following transplant. The remaining variables of age at transplant, donor age, gender, ECOG status, and disease risk index were not independently associated with survival in this sample.

**Table 2:** Survival analysis for individual predictors and multivariable survival analyses

	Individual Predictors		Multivariable Model	
Predictor	HR (95% CI)	*p*-value	Adjusted HR (95% CI)	*p*-value
Donor age	0.99 (0.93–1.06)	0.801		
Male gender	1.55 (0.64–3.76)	0.328		
ECOG	1.51 (0.57–4.05)	0.337		
Age at transplant	1.09 (0.98–1.20)	0.134	1.13 (1.00–1.27)	0.050
Comorbidity index	1.17 (1.01–1.36)	0.032	1.05 (0.89–1.24)	0.575
Disease risk index	3.03 (0.70–13.07)	0.137	2.62 (0.62–11.1)	0.190
REBOUND enrollment	0.316 (0.12–0.82)	0.017	0.302 (0.10–0.87)	0.027

*HR* Hazard Ratio, *CI* Confidence Interval

To explore mutually adjusted relationships in our small sample, multivariate Cox modeling for survival analysis was performed with age at transplant, comorbidity index, disease risk index, and REBOUND enrollment since these predicted survival with *p* < 0.25 [13–15] and highly clinically relevant to survival. Table 2 displays results of this multivariable model. When controlling for these covariates, mortality hazard was decreased by 70% among REBOUND participants compared to those declining enrollment (HR 0.302, 95% CI 0.10–0.87, *p* = 0.027). To further explore the impact that social determinants of health may have on survival; gender and race were added to the model and hazards of death based on REBOUND enrollment was relatively unchanged (HR 0.29, 95% CI 0.1–0.84, *p* = 0.022; not displayed).

## Discussion

We found that older adults who decided to enroll in a non-therapeutic alloBMT study had higher post-transplant survivorship than those who declined enrollment. This improved survival was noted to maintain when controlling for relevant clinical and social factors that influence survival post-transplant. This non-therapeutic study offers no intervention that could plausibly improve survival, so we surmise that the factors that drive the decision to participate in research also influence important clinical outcomes and participants who enrolled in this study were, at baseline, more likely to survive their disease than those who declined enrollment. It appears there are unknown factors that influence study enrollment which also impact survival after transplant that are presently unidentified.

All participants included in this evaluation received allogeneic transplant for a hematological malignancy and underwent non-myeloablative transplant preparation, which allows for some clinical uniformity in this participant sample. Disease risk index was used in these analyses to add additional control for clinical severity of the underlying diagnosis requiring transplant and comorbidity index was utilized to account for additional health factors that are known to impact transplant survival. Unfortunately, the sample size in this study is not adequate to appropriately stratify survival analyses based on categorical variables, and therefore type of transplant was not included in adjusted survival models. However, there were comparable proportions of those who received matched unrelated transplant (6 enrolled in the parent study, 5 declined) as well as haploidentical transplants (23 enrolled, 21 declined) despite differences in matched related and mismatched unrelated donors, but these sizes were small (*n* = 4 each).

Participants in this study were noted to be predominantly White (85.7%) with relatively high ECOG performance status, low comorbidity scores, and from census tracts that were not designated as low income (84.1%) and therefore had relatively low neighborhood poverty rates. These characteristics were not different between those who elected or declined enrollment in the parent REBOUND study and since all patients undergoing transplant were included in these analyses, this finding is likely not related to study selection bias but rather is reflective of the fact that these are the types of individuals with hematologic malignancies that undergo transplant. While minority groups bear a disproportionate burden of cancer diagnoses in the United States [16, 17], persons from low-income and marginalized groups have lower rates of AlloBMT [18]. The relatively affluent and predominantly White characteristics of this study sample likely reflects that these are the individuals who are referred for transplant or have resources that can be mobilized to access transplant services. National trends in allogeneic transplant for acute myeloid leukemia and myelodysplastic syndrome are 74% and 82% White, respectively [19], suggesting that this patient sample is reflective of the national transplant population. Higher performance status is also expected with older adult patients because transplant eligibility is more stringent compared to younger patients due to expected higher non-relapse mortality. Given that these clinical demographics are similar to those for transplant recipients nationally, findings from this small analysis can confidently be interpreted as initial accurate observations for the target population of this study.

Participation in well-designed clinical trials is an important good that should be widely available. It provides access to relevant novel therapy, is recommended by all major clinical societies, and informs the care of all persons with cancer. However, it is presently understood that clinical trial participants do not represent the overall oncology population [4, 5], including those specific to older adults. This study illustrates that differences between those who elect to participate in clinical studies and those who do not may also influence survival, even when study and non-study groups have similar demographic and clinical profiles. This research approach should be conducted with a larger and more-diverse set of clinical institutions or with cancer registry databases to further explore these relationships and confirm these findings. Further work should be conducted to discover which of the unidentified factors may be driving the decision to enroll in clinical studies and to evaluate if these also influence transplant survival and have relation between those who do and do not ultimately undergo transplantation.

This study is limited by its comparative design which precludes ability to draw causative relationships between factors associated with enrollment in REBOUND. Clinician bias also influences enrolling persons on oncology studies [5] and cannot be ruled out; however, enrollment was offered to all eligible patients in this parent study, so this bias is believed to be minimized. The sample size for this analysis is small and so our findings should be considered exploratory and statements of no association cannot confidently be made, but this work sets foundation for larger future studies to better explore these phenomenon.

## Conclusions

Understanding factors that influence enrollment in studies may help improve outcomes for all persons with cancer and facilitate targeted interventions to increase broad participation. These data provide new insight into the characteristics of older research participants and highlight that even when demographically similar, research groups may still be distinct from the study population at large. Results from clinical studies should be interpreted with the understanding that research participants may have higher clinical function and higher chance of survival at baseline and concerted efforts should be made to improve understanding and enrollment of all persons in research studies.

## Data Availability

The data used in this study are available from the corresponding author on reasonable request.
